# From Beauty to Protection: How Phenotypic Traits Influence Conservation Perceptions of Freshwater Fish

**DOI:** 10.3390/ani16111696

**Published:** 2026-06-01

**Authors:** Jana Fančovičová, Simona Todáková, Pavol Prokop

**Affiliations:** 1 Faculty of Education, Trnava University, Priemyselná 4, 91843 Trnava, Slovakia; jana.fancovicova@truni.sk (J.F.); simona.todakova@tvu.sk (S.T.); 2 Department of Environmental Ecology and Landscape Management, Faculty of Natural Sciences, Comenius University, Ilkovičova 6, 84215 Bratislava, Slovakia; 3 Institute of Zoology, Slovak Academy of Sciences, Dúbravská cesta 9, 84506 Bratislava, Slovakia

**Keywords:** fish attractiveness, flagship species, freshwater biodiversity, public perception, willingness to protect

## Abstract

Biodiversity conservation is closely linked to human willingness to conserve individual species. However, fish, as the least visible group of vertebrates, are often overlooked, despite the fact that they constitute almost 60% of all known vertebrate species and are characterized by great morphological and color diversity. Our study aimed to identify phenotypic traits of freshwater fish that influence their attractiveness and, at the same time, the perceived need for their conservation. The research consisted of two parts: a touchscreen-based evaluation of 30 fish species followed by an online survey focusing on a selected subset of 15 species. The most attractive species were those with colorful coloration, unusual body shape and distinctive fins. Respondents also attributed a high conservation value to species with striking or unusual features. An interesting finding was that even less attractive species were able to arouse a willingness to protect them thanks to their unusual appearance, which evoked prehistoric signs and aroused a feeling of compassion. Only species with serpentine body shapes received the lowest rankings in both attractiveness and willingness to protect. Based on these findings, we recommend using attractive and atypical fish species as so-called flagship species, which can effectively support the protection and preservation of the biodiversity of freshwater ecosystems. The results also represent a starting point for the creation of educational programs that can shape positive public attitudes towards fish and their conservation.

## Simple Summary

This study examined how phenotypic traits of freshwater fish shape perceived attractiveness and willingness to protect them. The research consisted of two parts: a touchscreen-based evaluation of 30 fish species and a subsequent online survey focusing on a selected subset of 15 species. The results showed that fish with conspicuous visual traits, especially colorful coloration, unusual body shape, and distinctive fins, were generally rated as the most attractive. Respondents also expressed greater willingness to protect species with striking or atypical features. Importantly, attractiveness was not the only factor associated with conservation support. Some less visually appealing species also evoked willingness to protect them, likely due to perceived rarity, vulnerability, or evolutionary uniqueness. One exception is species with serpentine body shapes, which are rated least attractive and unworthy of protection. Overall, the findings indicate that both beauty and distinctiveness influence conservation perceptions of fish. Visually appealing and atypical fish species may serve as effective flagship species in educational and conservation programs aimed at strengthening public support for freshwater biodiversity.

## 1. Introduction

The protection of biodiversity and its constant degradation is becoming an increasingly urgent problem, directly related to survival on Earth [1]. Many organisms are now on the verge of extinction, which is often a consequence of human reluctance or active persecution [2,3]. Human willingness to protect individual species is crucial for sustainable management of natural resources [4].

Scientists are currently intensively studying the question of which factors influence people’s willingness to protect organisms [4,5]. Conservation is often associated with iconic species such as pandas or elephants, suggesting that the size and attractiveness of the species may play a significant role [6]. In the case of fish, it has already been shown that human perception can determine the effectiveness of conservation programs [7], but so far there are no studies that would systematically examine public attitudes towards fish as a group. Lack of knowledge and low interest represent a significant obstacle even in the protection of endangered species. Fish, as the least visible group of vertebrates, remain perceived as less attractive compared to mammals or birds, while the negative assessment may be related to their morphological features, such as their serpentine body shape, reduced eyes or dull coloration [8]. Recent analyses showed that fish receive significantly lower attention than arachnids or insects, not only in YouTube videos [9] but also from editors who depict fish less frequently on journal cover pages and from researchers who publish fewer papers about fish than invertebrates in conservation journals [10]. Similarly, in other animals such as birds, body shape or proportions have been shown to significantly influence the assessment [11], while colour is more important only in some groups, such as parrots [12]. However, fish represent an extremely diverse group with more than 36,000 described species, i.e., approximately 60% of all vertebrates, which highlights their importance and the need for effective conservation.

The aim of our work was to find out which phenotypic traits of freshwater fish influence their attractiveness and at the same time contribute to the public’s willingness to protect them. The public’s attitudes, as well as the attitudes of young people, towards different species of organisms are different. Different preferences may have their origins in evolution. Many studies suggest that the attractiveness of animals can influence the perception of species, and consequently the willingness to protect them. In general, visually attractive and charismatic animal species that are close to humans are preferred, while unattractive or less well-known species, such as fish, often remain out of public interest. It is therefore important to identify the characteristics of fish that influence their perceived attractiveness/unattractiveness. It is also necessary to find out whether the perception of attractive traits will influence the willingness to protect fish. We hypothesized that respondents would rate fish with conspicuous phenotypic traits as more attractive than fish with inconspicuous phenotypic traits, and we also hypothesized that fish that respondents would rate as more attractive would be willing to protect more than fish that they would rate as less attractive.

We divided the research into two parts. In the first part, we used a touch-screen method to obtain assessments of the attractiveness and willingness to protect selected freshwater fish species using Matrix software. The study consisted of two consecutive parts using different sets of fish species. In the first part, 30 species were evaluated. Based on these results, a subset of 15 species was selected for the second part (online survey). Therefore, different species may appear across individual parts of the study. This touch-screen approach has been successfully used to examine threat detection in humans and human preferences for animals in the context of conservation priorities [13]. We assumed that respondents would evaluate fish with striking phenotypic traits differently than those with less striking phenotypic traits.

The aim of the second partial part of the research was to find out, through online questionnaires, which of the selected freshwater fish species are attractive/unattractive to students, and which of them they want to protect more/less. It was also part of the study to find out and subsequently evaluate which of the mentioned phenotypic traits of fish influence the assessment of their attractiveness and willingness to protect them.

## 2. Materials and Methods

### 2.1. Participants

The research with touchscreen method involved 42 respondents (33 women and 9 men), aged 22 to 24. This was a convenience sample of university students. The respondents in this part of the research were divided into two groups. The first group of participants was instructed to find the most attractive species, and the second group was instructed to find the species they would be most willing to protect. Both groups were provided with the same set of images. Each respondent participated in the research voluntarily. None of the participants were familiar with the hypotheses.

N = 203 respondents participated in the online research on the assessment of fish attractiveness (Table S1 and File S1). The research sample consisted of 138 (68%) women and 65 (32%) men. The research involved students who were currently attending primary school or high school. Of these, 116 (57%) students attended primary school and 87 (43%) students attended high school. The age range was between 9 and 19 years. When asked whether the respondents or someone in their family was engaged in fishing, we found that only 54 respondents answered positively, which represents 27% of the entire sample of research participants.

N = 195 respondents participated in the online research on willingness to protect fish. The research sample consisted of 113 (58%) women and 82 (42%) men (Table S2 and File S1). Students who were currently attending primary school or grammar school participated in the research. Of these, 92 (47%) students were attending primary school and 103 (53%) students were attending grammar school. The age structure of the respondents was between 10 and 18 years. When asked whether the respondents or someone in their family was engaged in fishing, we found that only 49 respondents answered positively out of all the participants, which represents 25% of the entire sample of research participants.

### 2.2. Photographs as Visual Stimuli

**Figure 1:**
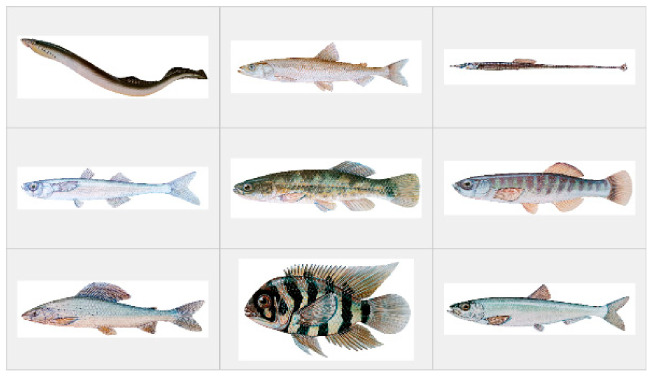
Generated images using a 3 × 3 grid in Matrix software.

For the touchscreen research, we prepared a set of 30 images of freshwater fish species. The initial set of 30 fish species was selected to represent a wide range of morphological and visual diversity, including variation in body shape, coloration, and distinctive features such as fins, spines, or body patterns. Although most species were European freshwater or brackish fish, a small number of non-native or tropical species were included to increase variability in phenotypic traits and enhance the robustness of the analysis. We randomly selected one specific species from one family so that the selected fish species covered a wide morphological variability, including aposematic features such as spines, whiskers, colored spots or stripes, large eyes, and different body shapes. We then edited all photos into a standardized form using the online application PhotoRoom Studio Photo Editor (https://www.photoroom.com, accessed on 1 April 2026). We edited the fish photos to the same position, comparable body size, and placed them on a white background. Respondents selected a target image of a fish, repeating the above procedure ten times (each time with a differently generated grid of 9 fish) (Figure 1). A higher number of clicks for a given fish characterized higher attractiveness and higher willingness to protect.

For the second part of the touchscreen research, we selected photographic material based on the results of the previous first part of the research conducted using the Matrix software (https://www.matrix-software.com/). From the 30 evaluated freshwater fish species, we filtered out 15 fish species in such a way that from the resulting ranking of the evaluated fish in the Matrix, we selected 5 fish species that were rated as the most attractive, 5 fish species that achieved an average attractiveness score, and 5 fish species that were rated as the least attractive. The research participants rated the extent to which the fish in the picture was attractive to them/to what extent they would like to protect the fish in the picture using a 5-point Likert scale. A higher score indicated higher attractiveness and a higher willingness to protect. They then justified each assessment in an open-ended question.

### 2.3. Procedure

We conducted the touchscreen research during the months of November–December 2023 and January 2024. We ensured that the research was carried out in such a way that the composition of the first and second groups of respondents was different from each other. The first group of respondents searched for the fish that was most attractive to them among the displayed species. The task of the second group was to find the fish that they wanted to protect the most.

The online research was conducted from October 2024 to February 2025. One questionnaire focused on ratings of fish attractiveness, and the other (identical version) focused on willingness to protect fish. We collected data over a period of five months, with both questionnaires being freely available to all respondents at the web addresses. Respondents’ responses were recorded directly in Google Forms. Participants were asked for their demographic information (school type, age, gender) and then to analyse their reactions to a series of images of freshwater fish. For each image, participants rated the fish on attractiveness (N = 203) or willingness to protect (N = 195) using a Likert scale (e.g., 1 = not at all attractive, 5 = extremely attractive).

### 2.4. Statistical Analyses

We examined whether data from online questionnaires were suitable for factor analysis. Since Bartlett’s test of sphericity was significant for both attractiveness (χ^2^ = 477, df = 66, *p* < 0.001) and willingness to protect (χ^2^ = 546, df = 91, *p* < 0.001), and the KMO values were 0.745 and 0.794, respectively, the data were suitable for exploratory factor analysis. Exploratory factor analysis (EFA) with Oblimin rotation was used for statistical processing of the data. Factor loadings indicate the strength of association between each species and the respective factor, while higher values represent stronger relationships. We also applied an analogous data processing procedure within the analysis related to fish protection. Before the statistical analysis itself, the average score of each fish of each respondent was first calculated (Tables S1 and S2).

We examined the effects of the predictors (age, gender, and fishing) on mean scores derived from ratings of attractiveness and willingness to protect fish (based on EFA-derived clusters; dependent variables) using a series of generalized linear models (GLMs) with a gamma distribution and log link function. Analyses were conducted in jamovi [14].

### 2.5. Word Analyses

All respondents’ answers to the open-ended question in the questionnaire “Justify why you find a given fish attractive” were divided into several categories according to their content, such as appearance, beauty, scales, fins, coloration, whiskers, etc. Based on the data processed in this way, a visual representation was created for each fish using the WordArt editor, supplemented with a brief verbal description of the most common reasons for perceiving the attractiveness of a given fish.

All respondents’ answers to the open-ended question in the questionnaire “Justify why you would like to protect the given fish” were divided into several categories according to their content, such as food, rarity, commonness, appearance, beauty, fear, etc. Based on the data processed in this way, a visual representation was created for each fish using the WordArt editor, supplemented with a brief verbal description of the most common reasons for willingness to protect the given fish.

## 3. Results

### 3.1. Fish Attractiveness

Before the actual statistical analysis, the average score of each fish for each respondent was first calculated. The data were then subjected to an exploratory factor analysis. The average score of fish attractiveness ratings that loaded on more than one factor and had a factor score greater than or equal to 0.3 were removed, and the corresponding species were excluded from the final analysis. The EFA was then run again until we reached a state of unique factors with fish that did not load on more than one factor. The analysis divided the scores into three factors (groups) (Table 1).

**Figure 2:**
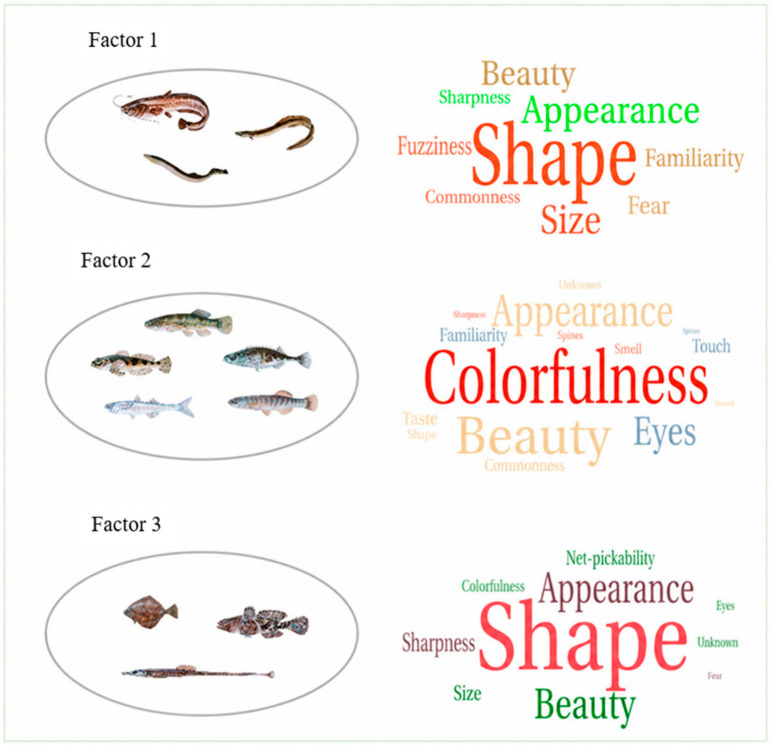
Visual classification of fish into groups based on attractiveness (EFA).

Overall, the factors explained 35.9% of the variability in the results (Table 2). Factor 1 was represented by fish with a serpentine body shape such as the *A. anguilla*. Factor 2 was represented by fish with a typical body shape such as the *U. krameri* and Factor 3 was represented by fish with atypical shapes such as the *P. flesus* (Figure 2).

We then analyzed the effects of the predictors (age, gender, and fishing) on mean scores for the three dependent variables (Factors 1–3).

**Figure 3:**
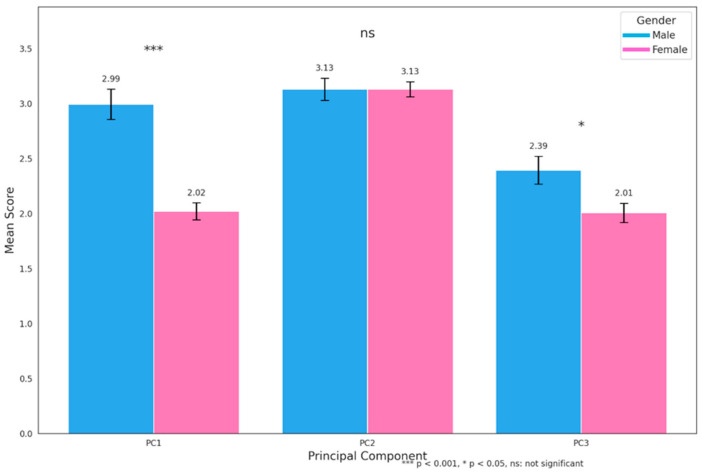
Differences in the average score of the three groups of fish with respect to the gender of the respondents.

We found that when analyzing the attractiveness of fish in the Factor 1 group, only the effect of gender was significant (Table 3 and Table S3). The attractiveness of fish was rated significantly lower in women than in men (Figure 3). Age of respondents and fishing were not associated with fish attractiveness scores.

When analyzing the influence of factors on the Factor 2 group of fish, we found that none of the factors were significant (Table S4). For the Factor 3 group of fish, the same trend was found as in the Factor 1 group of fish (Table S5). Women had significantly lower fish attractiveness scores than men (Figure 3). Interactions between gender and fishing were never significant, and subsequent exclusion of fishing, which is never significant, did not affect the results of the models.

### 3.2. Fish Protection

Before statistical analysis, the average score of each fish for each respondent was first calculated. The results were subjected to exploratory factor analysis. The average score of the fish protection assessment that loaded on more than one factor and had a factor score greater than or equal to 0.3 was removed, and the corresponding species were excluded from the final analysis. The EFA was thus run again until we reached a state of unique factors with fish that did not load on more than one factor. The analysis finally divided the scores into three factors (groups) (Table 4).

Overall, the factors explained 36.9% of the variability in the results (Table 5).

**Figure 4:**
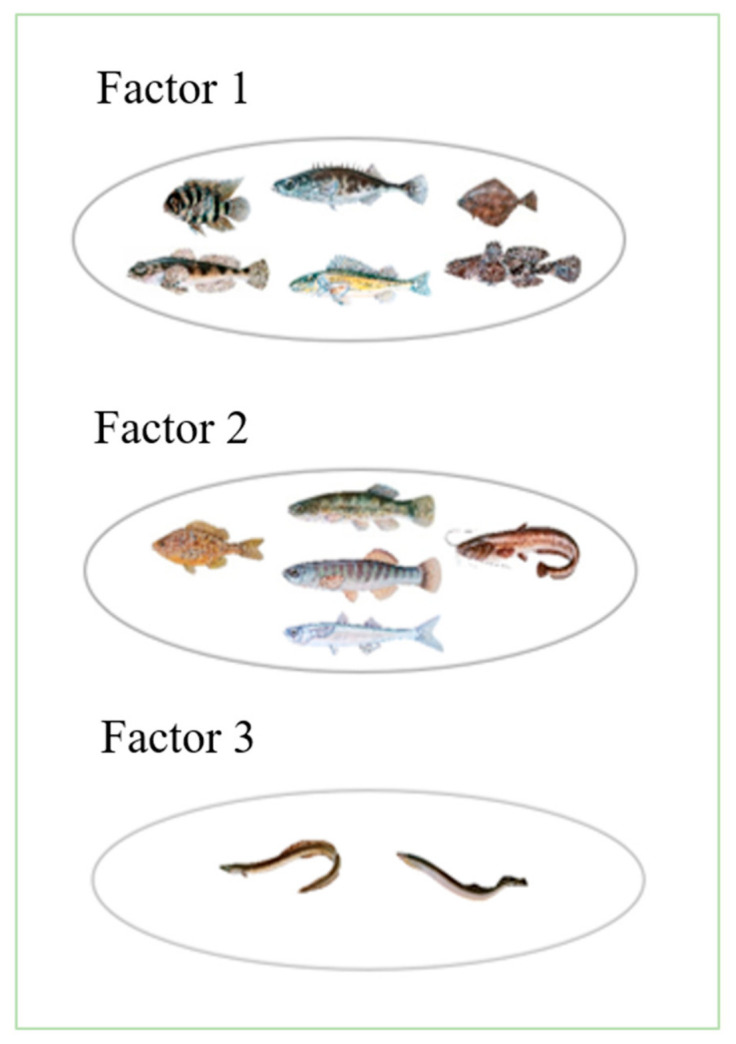
Visual classification of fish into conservation groups (EFA).

Factor 1 was represented by fish with atypical body shapes such as the *A. facetum*, *G. schraetser*, *P. flesus*, *C. poecilopus*, *G. aculeatus*, and the *G niger*. Factor 2 was represented by fish with typical body shapes such as the *U. krameri*, the *S. glanis*, the *L. gibbosus*, *M. praecox*, *A. fasciatus*, and Factor 3 was represented by species with a serpentine body shape such as the *A. anguilla*, the *C. wagneri* (Figure 4).

It should be noted that the factor labels used here describe the dominant visual characteristics of species grouped together by the exploratory factor analysis and should not be interpreted as fixed morphological categories. Because the attractiveness and protection analyses were based on different response variables, some species were associated with different factors in the two analyses. For example, *S. glanis* was grouped with serpentine-bodied species in the attractiveness analysis but with more typical-bodied species in the protection analysis, whereas *C. poecilopus* shifted from the typical-bodied group in the attractiveness analysis to the atypical-bodied group in the protection analysis. These shifts likely reflect the intermediate or visually ambiguous morphology of these species and show that respondents may rely on different visual cues when judging attractiveness versus conservation priority.

The next step was to analyze predictors such as age, gender and fishing, which were used as independent variables. The dependent variables were always the average scores of individual factors. We found that none of the factors were significant in all three groups when analyzing the fish protection rating (Tables S6–S8).

## 4. Discussion

In the present research, we focused on identifying the characteristic features of freshwater fish that contribute to their attractiveness and those that may contribute to their conservation. By analyzing the data related to attractiveness, we divided the fish into three groups based on their morphological features. The first group consisted of fish with a serpentine body shape, the second group consisted of fish with conspicuous visual traits such as colorful body coloration, spiny fins, and distinctive body patterns, and the third group consisted of fish with darker coloration and atypical body shape. We found that the most attractive species were species from the second group and were characterized by conspicuous coloration, unusual body shape, and distinctive fins, such as *M. praecox*, *U. crameri*, and *A. faciatus*. *A. fasciatus* males have blue-grey vertical bars on the flanks and yellow-green fins, which might be considered visually pleasing. *M. praecox* also ranked highly (3rd) among the species requiring protection. In this case, perceived attractiveness correlated with the need for protection. We suggest that the positive evaluation of this blue-silver species may be explained by its bright, reflective coloration. Blue and silver tones are common in many open-water fish and may be perceived as clean, vivid, and visually attractive. In addition, silvery or glossy surfaces may attract human attention because they resemble the sparkle or reflection of water. This interpretation is consistent with Meert et al. [15], who showed that humans tend to prefer glossy objects and argued that this preference may stem from an evolved sensitivity to visual cues associated with fresh water. Thus, the attractiveness of blue-silver fish may reflect a broader human affinity for shiny, water-like visual features rather than a response to unusual or imaginary appearance. 

The need for protection showed somewhat different patterns than attractiveness. The first and second groups of fish were represented by species with atypical and typical body shapes, respectively. The third group comprised species with a serpentine body shape. Although *S. nigrolineatus* was removed from factor analyses, it should be noted that it ranked highest, probably reflecting the need for seahorse conservation [16]. *A. facetum* ranked high in all probability due to its conspicuous coloration, which could attract participants’ attention.

These findings can be compared with the results reported by Lipták et al. [5], who also examined perceived attractiveness and willingness to protect freshwater taxa. Their study showed that attractiveness was positively associated with conservation support. Our data also suggest that visual appeal and distinctive external features play an important role in shaping conservation-related attitudes toward fish. At the same time, the comparison also suggests that conservation judgments are not determined exclusively by beauty. Some species may evoke support for protection because they are perceived as rare, unusual, threatened, or biologically interesting, even if their attractiveness is not among the highest. Unlike Lipták et al. [5], our study used independent participant groups for attractiveness and willingness-to-protect ratings. Therefore, our findings should be interpreted as a species-level association between independently obtained evaluations, rather than as evidence that the same individuals who perceived a species as attractive were also more willing to protect it. This design reduces possible carryover or consistency effects between rating domains, although it does not permit direct individual-level inference.

Certain species were assigned to different morphological categories across analyses (e.g., *S. glanis*, *C. poecilopus*), which may reflect the fact that their body shapes are intermediate between categories. This suggests that certain phenotypic traits may be perceived differently depending on the evaluative context (attractiveness vs. conservation). Respondents expressed a greater willingness to protect species with striking or unusual features. At the same time, even less visually attractive species evoked willingness to protect them, likely due to their perceived rarity, vulnerability, or evolutionary uniqueness.

The inclusion of fish with preferred phenotypic traits as flagship species has been shown to be an effective tool that can effectively contribute to their conservation even among the wider public [17]. This is crucial given that fish are highly ignored by both scientists and the general public [9,10]. Our results show that the principles shaping the willingness to protect fish may not necessarily be based solely on aesthetic appeal. Attractive fish species should not be the only candidates selected as flagship species. For instance, *S. nigrolineatus* ranked among the least attractive species yet first for protection need. Despite the fact that conservation campaigns have traditionally been dominated by attractive species [18], our findings show that even less attractive species can evoke positive conservation attitudes. Less attractive species may also play an important role in fish conservation. Due to their peculiar appearance, which resembles a prehistoric or ancient fish, they evoked a feeling of compassion in respondents, which made them willing to protect them. This effect may be caused precisely by their atypical “ancient” appearance, which triggers a feeling of vulnerability in people, as they perceive these species as endangered or evolutionarily unique [19]. Species perceived as endangered or evolutionarily unique trigger protective mechanisms in people based on empathy [20]. It seems that, unlike attractive species, which gain public support mainly through aesthetic perception, less attractive species may benefit from emotional reactions based precisely on the perception of their endangerment and compassion.

Some species were assigned to different factors in the attractiveness and protection analyses. This is not necessarily contradictory, because the two EFAs were based on different evaluations and the factor names represent post hoc perceptual labels rather than strict morphological categories. Species such as *S. glanis* and *C. poecilopus* have intermediate or visually ambiguous body forms, and respondents may therefore have emphasized different traits when judging attractiveness compared with conservation priority. This suggests that attractiveness and willingness to protect are related but not identical perceptions, and that different morphological cues may influence each judgement. 

However, species like *C. wagneri* and *A. anguilla* received low scores in both attractiveness and protection rankings. The serpentine body shapes of these species likely play a crucial role in their low rankings, perhaps because they resemble snakes—which exerted significant predation pressure in our evolutionary past [21]. Alternatively, they could resemble intestinal parasites like tapeworms, which evoke disgust due to disease associations [22]. For these two serpentine species, we do not recommend their use in conservation campaigns owing to associations with deep-seated psychological threats of predation and/or disease.

Our results indicate that visual and emotional factors are involved in shaping the perceived willingness to protect specific fish species, while the unusual morphology of a species, which at first glance appears unattractive, may not automatically negatively affect the conservation potential of the species. Such a mechanism, which draws attention to the uniqueness and vulnerability of a particular species, is particularly significant in the case of freshwater fish, which are often perceived by society as less valuable compared to terrestrial animals.

Langlois et al. [23] showed that the aesthetic value of reef fishes is mismatched with conservation priorities, with less attractive species often being more threatened and more evolutionary or ecologically distinct. Our results similarly suggest that visual traits shape willingness to protect freshwater fish. However, protection ratings were not simply a reflection of attractiveness: some atypically shaped species also received high conservation support. This suggests that both perceived beauty and distinctiveness may influence conservation attitudes, and that unusual-looking freshwater fishes could be useful flagship species if their ecological uniqueness is clearly communicated.

By including less attractive but ecologically important fish species as flagship species, it is possible to increase the general public’s knowledge of the threatened status of fish and consequently ensure the protection of less attractive but equally threatened species. This approach can help shape the overall perception of the importance of fish conservation and influence not only public attitudes towards fish, but also efforts to preserve the threatened biodiversity of freshwater ecosystems as a whole [24]. In the future, therefore, the question arises for conservation strategies with the intention of re-evaluating the traditional criteria for the selection of flagship species, not only for freshwater fish, but also for other species.

## Figures and Tables

**Table 1:** Distribution of attractiveness scores into three factors.

	Factor			
Type of Fish	1	2	3	Uniqueness
*Umbra krameri*		0.47		0.79
*Anguilla anguilla*	0.84			0.30
*Platichthys flesus*			0.51	0.69
*Silurus glanis*	0.45			0.75
*Cottus poecilopus*		0.52		0.69
*Gasterosteus aculeatus*		0.50		0.59
*Caspiomyzon wagneri*	0.75			0.42
*Melanotaenia praecox*		0.51		0.74
*Gobius niger*			0.45	0.67
*Aphanius fasciatus*		0.57		0.70
*Syngnathus nigrolineatus*			0.67	0.52

Note: “Uniqueness” represents the distinctiveness of species within each factor based on their factor loadings.

**Table 2:** Variability of attractiveness factors.

Factor	% of Variance	Cumulative %
1	13.76	13.80
2	12.95	26.70
3	9.19	35.90

**Table 3:** Influence of factors on the assessment of fish attractiveness of Factor 1.

Parameters	Coef	z	*p* > |z|
Gender	−0.41	−6.50	0.001
Fishing	0.11	1.60	0.11
Age	−0.02	−1.73	0.08

**Table 4:** Distribution of protection scores into three factors.

	Factor			
Type of Fish	1	2	3	Uniqueness
*Umbra krameri*		0.35		0.85
*Australoheros facetum*	0.68			0.50
*Anguilla anguilla*			0.90	0.20
*Gymnocephalus schraetser*	0.69			0.55
*Platichthys flesus*	0.47			0.74
*Silurus glanis*		0.34		0.84
*Cottus poecilopus*	0.59			0.62
*Gasterosteus aculeatus*	0.46			0.72
*Lepomis gibbosus*		0.35		0.77
*Caspiomyzon wagneri*			0.38	0.80
*Melanotaenia praecox*		0.37		0.83
*Gobius niger*	0.64			0.56
*Aphanius fasciatus*		0,87		0.24

Note: “Uniqueness” represents the distinctiveness of species within each factor based on their factor loadings.

**Table 5:** Variability of protection factors.

Factor	% of Variance	Cumulative %
1	17.77	17.80
2	10.55	28.30
3	8.56	36.90

## Data Availability

Data are available as an electronic supplement.

## Supplementary Materials

The following supporting information can be downloaded at: https://www.mdpi.com/article/10.3390/ani16111696/s1, File S1: Table S1. Fish species attractiveness ratings ranked by mean Likert score; Table S2. Fish species protection ratings ranked by mean Likert score; Table S3. Generalized linear model regression results for fish attractiveness based on EFA-derived Factor 1 scores; Table S4. Generalized linear model regression results for fish attractiveness based on EFA-derived Factor 2 scores; Table S5. Generalized linear model regression results for fish attractiveness based on EFA-derived Factor 3 scores; Table S6. Generalized linear model regression results for willingness to protect fish based on EFA-derived Factor 1 scores; Table S7. Generalized linear model regression results for willingness to protect fish based on EFA-derived Factor 2 scores; Table S8. Generalized linear model regression results for willingness to protect fish based on EFA-derived Factor 3 scores.
