# Supplementary material for: From Beauty to Protection: How Phenotypic Traits Influence Conservation Perceptions of Freshwater Fish
Source: Animals (Basel). 2026 Jun 1;16(11):1696. doi: 10.3390/ani16111696 (PMC13255684; doi:10.3390/ani16111696)
Supplement: Supplementary file 1 [file animals-16-01696-s001.zip › animals-4267585-supplementary.pdf]

Supplementary Tables

Table S1. Fish species attractiveness ratings ranked by mean Likert score

Rank	Fish species	Mean	SD	N
1	*Melanotaenia praecox*	4.17	1.10	204
2	*Lepomis gibbosus*	3.80	1.26	204
3	*Umbra krameri*	3.43	1.06	204
4	*Aphanius fasciatus*	3.41	1.18	204
5	*Australoheros facetum*	3.33	1.32	204
6	*Silurus glanis*	2.95	1.48	204
7	*Cottus poecilopus*	2.84	1.19	204
8	*Gasterosteus aculeatus*	2.84	1.35	204
9	*Acipenser ruthenus*	2.75	1.32	204
10	*Gymnocephalus schraetser*	2.65	1.33	204
11	*Syngnathus nigrolineatus*	2.55	1.63	204
12	*Anguilla anguilla*	2.19	1.32	204
13	*Gobius niger*	2.07	1.34	204
14	*Caspiomyzon wagneri*	1.86	1.20	204
15	*Platichthys flesus*	1.78	1.17	204

Table S2. Fish species protection ratings ranked by mean Likert score

Rank	Fish species	Mean	SD	N
1	*Syngnathus nigrolineatus*	3.85	1.49	196
2	*Australoheros facetum*	3.77	1.29	196
3	*Melanotaenia praecox*	3.65	1.42	196
4	*Gymnocephalus schraetser*	3.60	1.32	196
5	*Platichthys flesus*	3.55	1.47	196
6	*Lepomis gibbosus*	3.55	1.27	196
7	*Acipenser ruthenus*	3.38	1.24	196
8	*Gasterosteus aculeatus*	3.36	1.24	196
9	*Gobius niger*	3.36	1.41	196
10	*Cottus poecilopus*	3.13	1.17	196
11	*Umbra krameri*	2.87	1.14	196
12	*Anguilla anguilla*	2.86	1.36	196
13	*Caspiomyzon wagneri*	2.62	1.36	196
14	*Aphanius fasciatus*	2.51	1.24	196
15	*Silurus glanis*	2.33	1.35	196

Table S3. Generalized linear model regression results for fish attractiveness based on EFA-derived Factor 1 scores.

**Parameter**	**β**	**SE**	**z**	**p-value**	**95% CI lower**	**95% CI upper**
Intercept	1.3937	0.195	7.132	<0.001	1.011	1.777
Sex: girl	-0.4111	0.063	-6.501	<0.001	-0.535	-0.287
Fishing experience: yes	0.1064	0.066	1.602	0.109	-0.024	0.237
Age	-0.0221	0.013	-1.730	0.084	-0.047	0.003

Note. β = regression coefficient; SE = standard error; CI = confidence interval; EFA = exploratory factor analysis. Reference categories are boy for sex and no fishing experience for fishing experience.

Table S4. Generalized linear model regression results for fish attractiveness based on EFA-derived Factor 2 scores.

**Parameter**	**β**	**SE**	**z**	**p-value**	**95% CI lower**	**95% CI upper**
Intercept	1.1737	0.122	9.624	<0.001	0.935	1.413
Sex: girl	-0.0012	0.039	-0.030	0.976	-0.079	0.076
Fishing experience: yes	-0.0028	0.041	-0.068	0.946	-0.084	0.078
Age	-0.0022	0.008	-0.272	0.786	-0.018	0.013

Note. β = regression coefficient; SE = standard error; CI = confidence interval; EFA = exploratory factor analysis. Reference categories are boy for sex and no fishing experience for fishing experience.

Table S5. Generalized linear model regression results for fish attractiveness based on EFA-derived Factor 3 scores.

**Parameter**	**β**	**SE**	**z**	**p-value**	**95% CI lower**	**95% CI upper**
Intercept	0.5399	0.227	2.380	0.017	0.095	0.984
Sex: girl	-0.1634	0.073	-2.230	0.026	-0.307	-0.020
Fishing experience: yes	-0.0599	0.077	-0.780	0.437	-0.211	0.091
Age	0.0238	0.015	1.600	0.109	-0.005	0.053

Note. β = regression coefficient; SE = standard error; CI = confidence interval; EFA = exploratory factor analysis. Reference categories are boy for sex and no fishing experience for fishing experience.

Table S6. Generalized linear model regression results for willingness to protect fish based on EFA-derived Factor 1 scores.

**Parameter**	**β**	**SE**	**z**	**p-value**	**95% CI lower**	**95% CI upper**
Intercept	1.0732	0.135	7.952	<0.001	0.809	1.338
Sex: girl	0.0075	0.039	0.192	0.847	-0.069	0.084
Fishing experience: yes	0.0213	0.043	0.491	0.623	-0.064	0.106
Age	0.0109	0.010	1.128	0.259	-0.008	0.030

Note. β = regression coefficient; SE = standard error; CI = confidence interval; EFA = exploratory factor analysis. Reference categories are boy for sex and no fishing experience for fishing experience.

Table S7. Generalized linear model regression results for willingness to protect fish based on EFA-derived Factor 2 scores.

**Parameter**	**β**	**SE**	**z**	**p-value**	**95% CI lower**	**95% CI upper**
Intercept	0.9117	0.135	6.762	<0.001	0.647	1.176
Sex: girl	0.0703	0.039	1.796	0.073	-0.006	0.147
Fishing experience: yes	-0.0256	0.043	-0.593	0.553	-0.110	0.059
Age	0.0106	0.010	1.098	0.272	-0.008	0.029

Note. β = regression coefficient; SE = standard error; CI = confidence interval; EFA = exploratory factor analysis. Reference categories are boy for sex and no fishing experience for fishing experience.

Table S8. Generalized linear model regression results for willingness to protect fish based on EFA-derived Factor 3 scores.

**Parameter**	**β**	**SE**	**z**	**p-value**	**95% CI lower**	**95% CI upper**
Intercept	1.2629	0.210	6.018	<0.001	0.852	1.674
Sex: girl	-0.0590	0.061	-0.968	0.333	-0.178	0.061
Fishing experience: yes	0.0492	0.067	0.731	0.465	-0.083	0.181
Age	-0.0161	0.015	-1.077	0.282	-0.046	0.013

Note. β = regression coefficient; SE = standard error; CI = confidence interval; EFA = exploratory factor analysis. Reference categories are boy for sex and no fishing experience for fishing experience.
